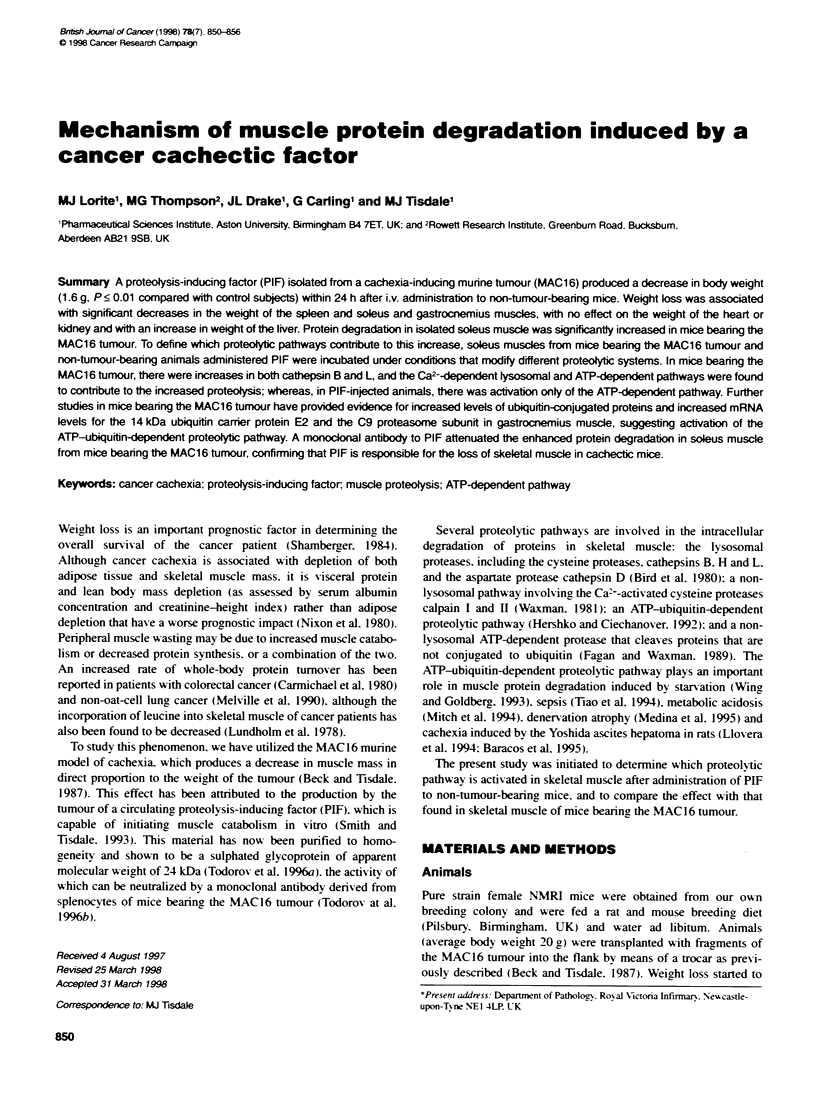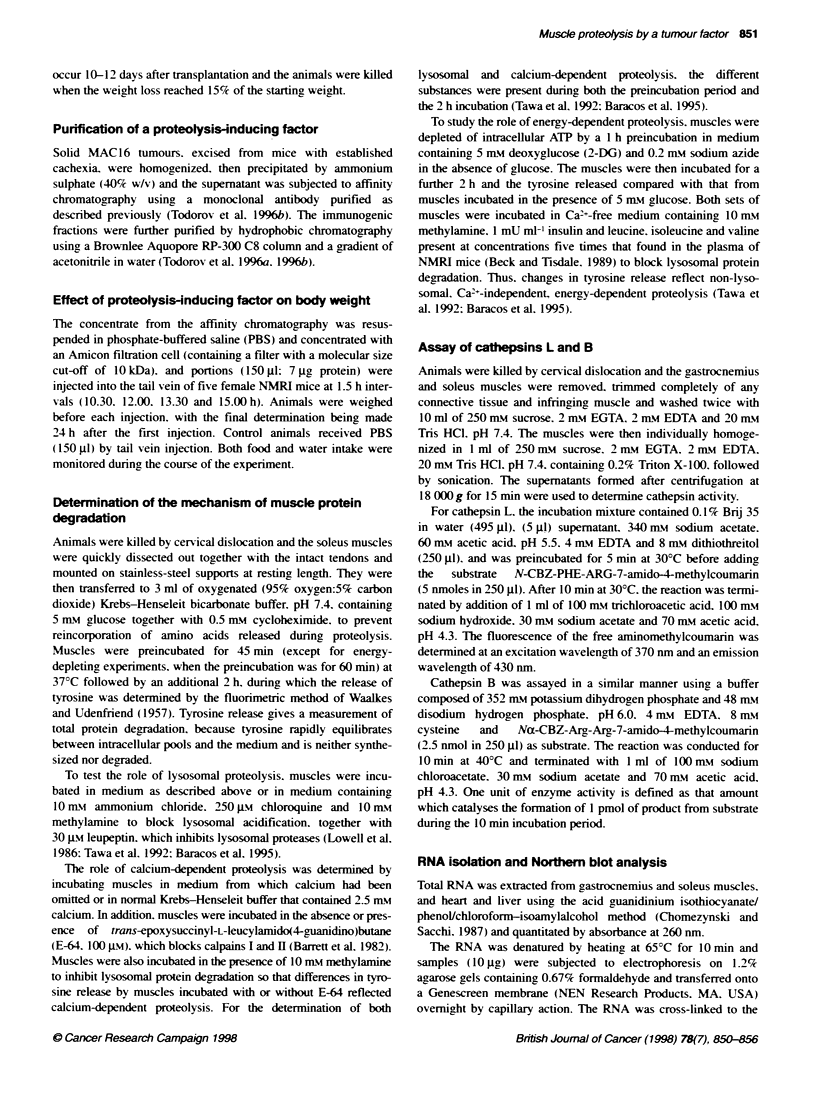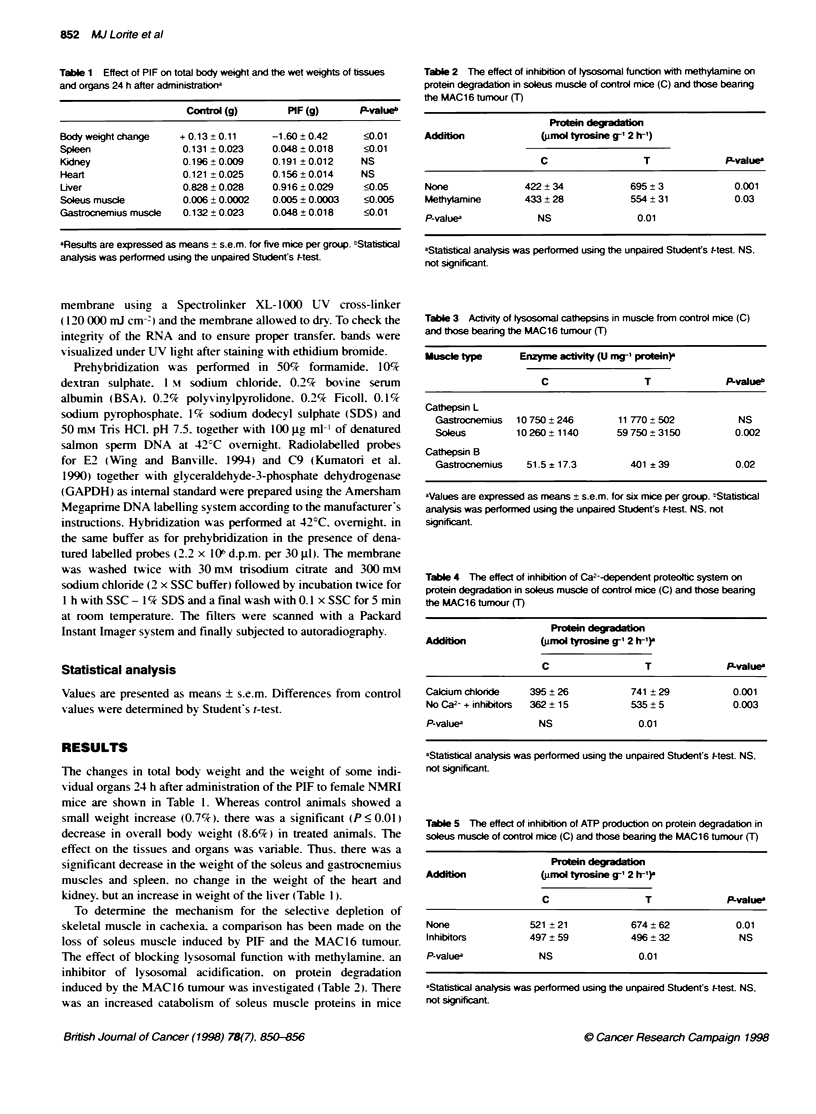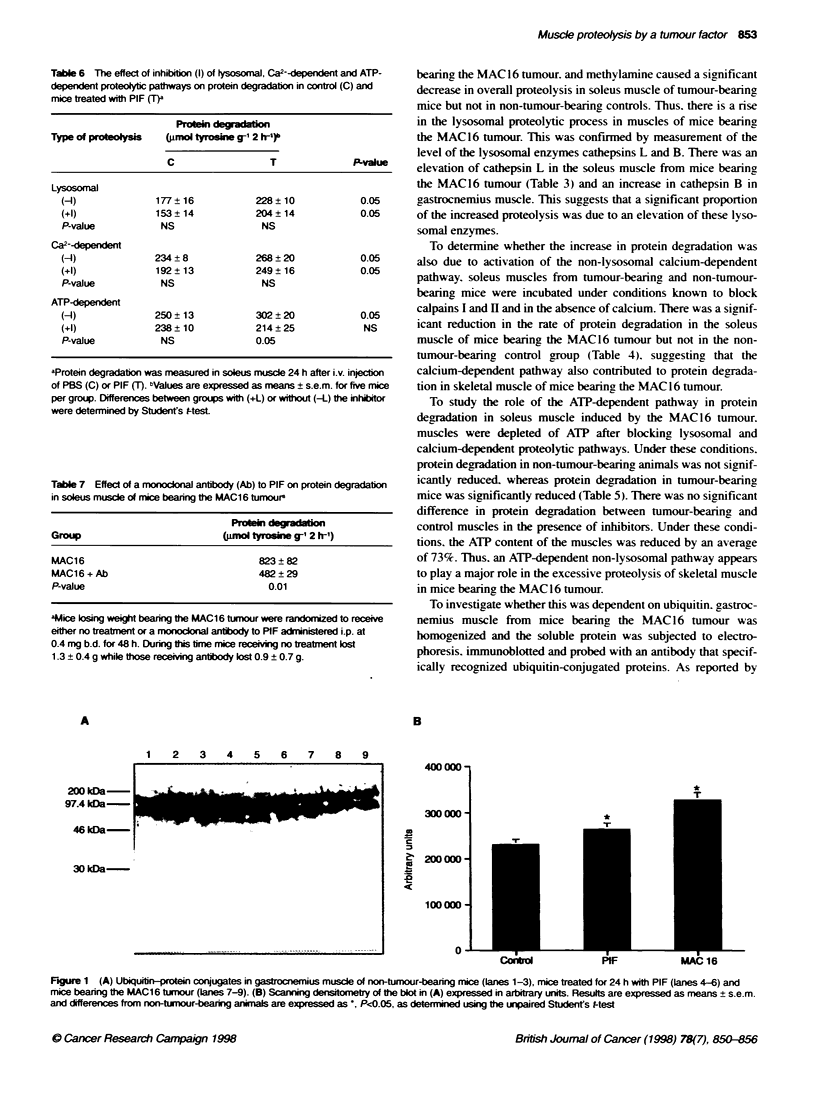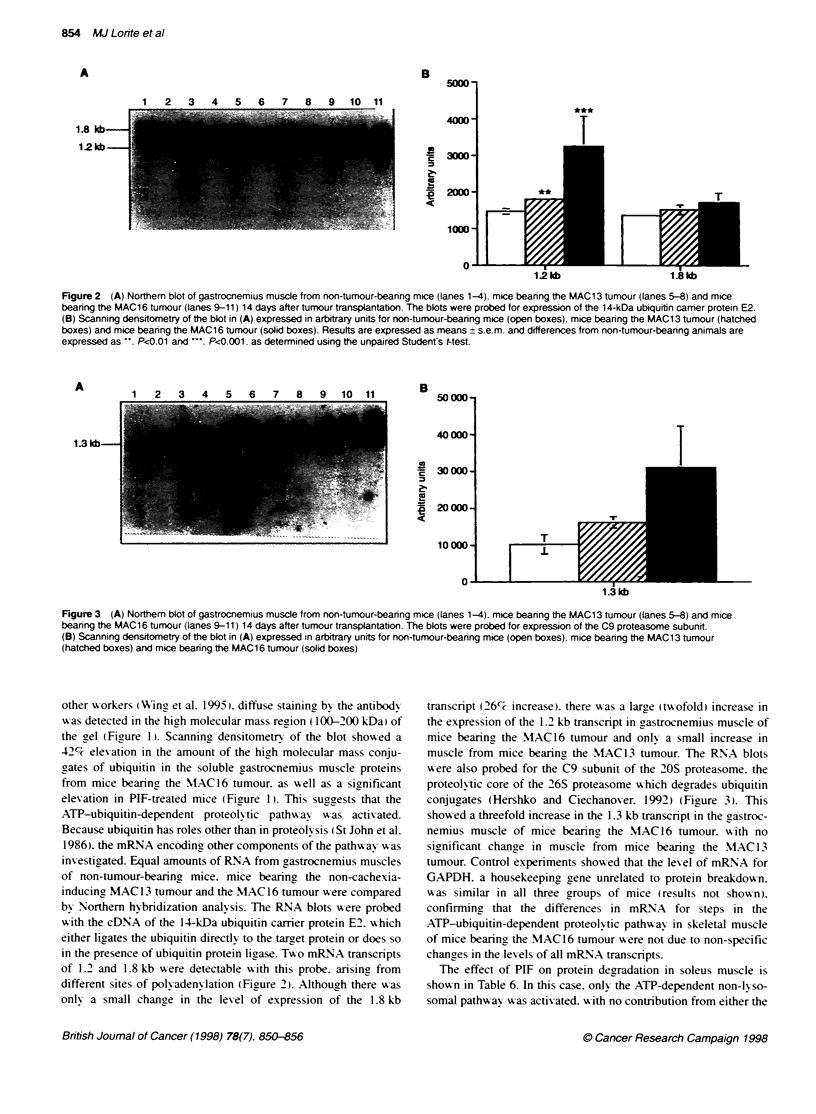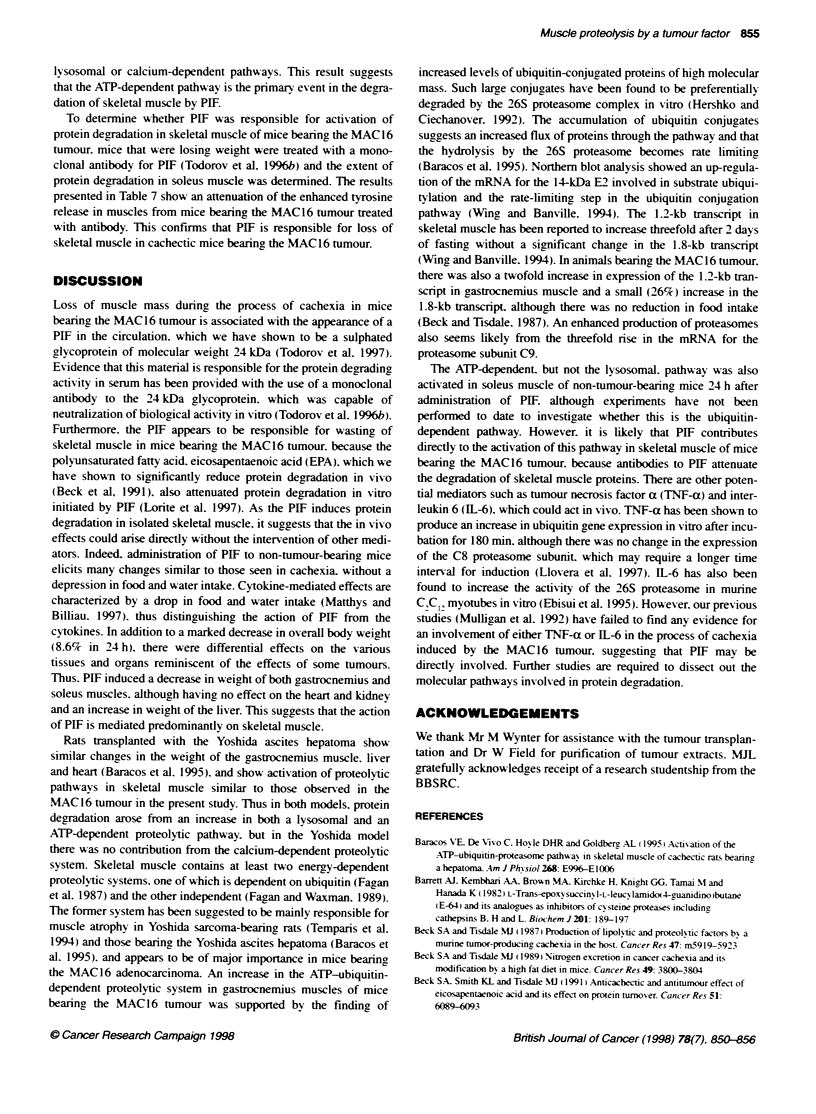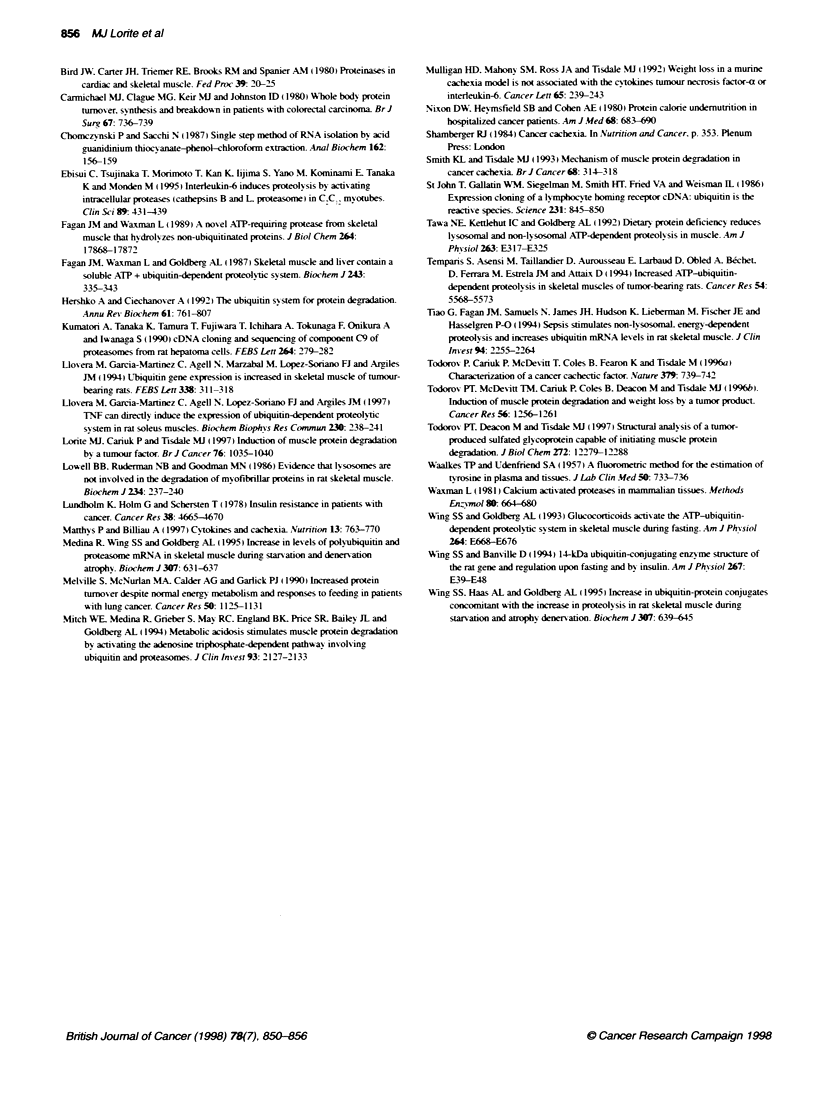# Mechanism of muscle protein degradation induced by a cancer cachectic factor.

**DOI:** 10.1038/bjc.1998.592

**Published:** 1998-10

**Authors:** M. J. Lorite, M. G. Thompson, J. L. Drake, G. Carling, M. J. Tisdale

**Affiliations:** Pharmaceutical Sciences Institute, Aston University, Birmingham, UK.

## Abstract

**Images:**


British Journal of Cancer (1998) 78(7), 850–856
© 1998 Cancer Research Campaign

### Mechanism of muscle protein degradation induced by a cancer cachectic factor

**MJ Lorite[1], MG Thompson[2], JL Drake[1], G Carling[1] and MJ Tisdale[1]**

[1]Pharmaceutical Sciences Institute, Aston University, Birmingham B4 7ET, UK; and [2]Rowett Research Institute, Greenburn Road, Bucksburn, Aberdeen AB21 9SB, UK

**Summary** A proteolysis-inducing factor (PIF) isolated from a cachexia-inducing murine tumour (MAC16) produced a decrease in body weight (1.6 g, $P \leq 0.01$ compared with control subjects) within 24 h after i.v. administration to non-tumour-bearing mice. Weight loss was associated with significant decreases in the weight of the spleen and soleus and gastrocnemius muscles, with no effect on the weight of the heart or kidney and with an increase in weight of the liver. Protein degradation in isolated soleus muscle was significantly increased in mice bearing the MAC16 tumour. To define which proteolytic pathways contribute to this increase, soleus muscles from mice bearing the MAC16 tumour and non-tumour-bearing animals administered PIF were incubated under conditions that modify different proteolytic systems. In mice bearing the MAC16 tumour, there were increases in both cathepsin B and L, and the $Ca^{2+}$-dependent lysosomal and ATP-dependent pathways were found to contribute to the increased proteolysis; whereas, in PIF-injected animals, there was activation only of the ATP-dependent pathway. Further studies in mice bearing the MAC16 tumour have provided evidence for increased levels of ubiquitin-conjugated proteins and increased mRNA levels for the 14 kDa ubiquitin carrier protein E2 and the C9 proteasome subunit in gastrocnemius muscle, suggesting activation of the ATP–ubiquitin-dependent proteolytic pathway. A monoclonal antibody to PIF attenuated the enhanced protein degradation in soleus muscle from mice bearing the MAC16 tumour, confirming that PIF is responsible for the loss of skeletal muscle in cachectic mice.

**Keywords:** cancer cachexia; proteolysis-inducing factor; muscle proteolysis; ATP-dependent pathway

Weight loss is an important prognostic factor in determining the overall survival of the cancer patient (Shamberger, 1984). Although cancer cachexia is associated with depletion of both adipose tissue and skeletal muscle mass, it is visceral protein and lean body mass depletion (as assessed by serum albumin concentration and creatinine–height index) rather than adipose depletion that have a worse prognostic impact (Nixon et al, 1980). Peripheral muscle wasting may be due to increased muscle catabolism or decreased protein synthesis, or a combination of the two. An increased rate of whole-body protein turnover has been reported in patients with colorectal cancer (Carmichael et al, 1980) and non-oat-cell lung cancer (Melville et al, 1990), although the incorporation of leucine into skeletal muscle of cancer patients has also been found to be decreased (Lundholm et al, 1978).

To study this phenomenon, we have utilized the MAC16 murine model of cachexia, which produces a decrease in muscle mass in direct proportion to the weight of the tumour (Beck and Tisdale, 1987). This effect has been attributed to the production by the tumour of a circulating proteolysis-inducing factor (PIF), which is capable of initiating muscle catabolism in vitro (Smith and Tisdale, 1993). This material has now been purified to homogeneity and shown to be a sulphated glycoprotein of apparent molecular weight of 24 kDa (Todorov et al, 1996*a*), the activity of which can be neutralized by a monoclonal antibody derived from splenocytes of mice bearing the MAC16 tumour (Todorov at al, 1996*b*).

*Received 4 August 1997*
*Revised 25 March 1998*
*Accepted 31 March 1998*

*Correspondence to:* MJ Tisdale

Several proteolytic pathways are involved in the intracellular degradation of proteins in skeletal muscle: the lysosomal proteases, including the cysteine proteases, cathepsins B, H and L, and the aspartate protease cathepsin D (Bird et al, 1980); a non-lysosomal pathway involving the $Ca^{2+}$-activated cysteine proteases calpain I and II (Waxman, 1981); an ATP–ubiquitin-dependent proteolytic pathway (Hershko and Ciechanover, 1992); and a non-lysosomal ATP-dependent protease that cleaves proteins that are not conjugated to ubiquitin (Fagan and Waxman, 1989). The ATP–ubiquitin-dependent proteolytic pathway plays an important role in muscle protein degradation induced by starvation (Wing and Goldberg, 1993), sepsis (Tiao et al, 1994), metabolic acidosis (Mitch et al, 1994), denervation atrophy (Medina et al, 1995) and cachexia induced by the Yoshida ascites hepatoma in rats (Llovera et al, 1994; Baracos et al, 1995).

The present study was initiated to determine which proteolytic pathway is activated in skeletal muscle after administration of PIF to non-tumour-bearing mice, and to compare the effect with that found in skeletal muscle of mice bearing the MAC16 tumour.

### MATERIALS AND METHODS

### Animals

Pure strain female NMRI mice were obtained from our own breeding colony and were fed a rat and mouse breeding diet (Pilsbury, Birmingham, UK) and water ad libitum. Animals (average body weight 20 g) were transplanted with fragments of the MAC16 tumour into the flank by means of a trocar as previously described (Beck and Tisdale, 1987). Weight loss started to

**Present address:* Department of Pathology, Royal Victoria Infirmary, Newcastle-upon-Tyne NE1 4LP, UK

occur 10–12 days after transplantation and the animals were killed when the weight loss reached 15% of the starting weight.

### Purification of a proteolysis-inducing factor

Solid MAC16 tumours, excised from mice with established cachexia, were homogenized, then precipitated by ammonium sulphate (40% w/v) and the supernatant was subjected to affinity chromatography using a monoclonal antibody purified as described previously (Todorov et al, 1996*b*). The immunogenic fractions were further purified by hydrophobic chromatography using a Brownlee Aquopore RP-300 C8 column and a gradient of acetonitrile in water (Todorov et al, 1996*a*, 1996*b*).

### Effect of proteolysis-inducing factor on body weight

The concentrate from the affinity chromatography was resuspended in phosphate-buffered saline (PBS) and concentrated with an Amicon filtration cell (containing a filter with a molecular size cut-off of 10 kDa), and portions (150 µl; 7 µg protein) were injected into the tail vein of five female NMRI mice at 1.5 h intervals (10.30, 12.00, 13.30 and 15.00 h). Animals were weighed before each injection, with the final determination being made 24 h after the first injection. Control animals received PBS (150 µl) by tail vein injection. Both food and water intake were monitored during the course of the experiment.

### Determination of the mechanism of muscle protein degradation

Animals were killed by cervical dislocation and the soleus muscles were quickly dissected out together with the intact tendons and mounted on stainless-steel supports at resting length. They were then transferred to 3 ml of oxygenated (95% oxygen:5% carbon dioxide) Krebs–Henseleit bicarbonate buffer, pH 7.4, containing 5 mM glucose together with 0.5 mM cycloheximide, to prevent reincorporation of amino acids released during proteolysis. Muscles were preincubated for 45 min (except for energy-depleting experiments, when the preincubation was for 60 min) at 37°C followed by an additional 2 h, during which the release of tyrosine was determined by the fluorimetric method of Waalkes and Udenfriend (1957). Tyrosine release gives a measurement of total protein degradation, because tyrosine rapidly equilibrates between intracellular pools and the medium and is neither synthesized nor degraded.

To test the role of lysosomal proteolysis, muscles were incubated in medium as described above or in medium containing 10 mM ammonium chloride, 250 µM chloroquine and 10 mM methylamine to block lysosomal acidification, together with 30 µM leupeptin, which inhibits lysosomal proteases (Lowell et al, 1986; Tawa et al, 1992; Baracos et al, 1995).

The role of calcium-dependent proteolysis was determined by incubating muscles in medium from which calcium had been omitted or in normal Krebs–Henseleit buffer that contained 2.5 mM calcium. In addition, muscles were incubated in the absence or presence of *trans*-epoxysuccinyl-L-leucylamido(4-guanidino)butane (E-64, 100 µM), which blocks calpains I and II (Barrett et al, 1982). Muscles were also incubated in the presence of 10 mM methylamine to inhibit lysosomal protein degradation so that differences in tyrosine release by muscles incubated with or without E-64 reflected calcium-dependent proteolysis. For the determination of both lysosomal and calcium-dependent proteolysis, the different substances were present during both the preincubation period and the 2 h incubation (Tawa et al, 1992; Baracos et al, 1995).

To study the role of energy-dependent proteolysis, muscles were depleted of intracellular ATP by a 1 h preincubation in medium containing 5 mM deoxyglucose (2-DG) and 0.2 mM sodium azide in the absence of glucose. The muscles were then incubated for a further 2 h and the tyrosine released compared with that from muscles incubated in the presence of 5 mM glucose. Both sets of muscles were incubated in $Ca^{2+}$-free medium containing 10 mM methylamine, 1 mU $ml^{-1}$ insulin and leucine, isoleucine and valine present at concentrations five times that found in the plasma of NMRI mice (Beck and Tisdale, 1989) to block lysosomal protein degradation. Thus, changes in tyrosine release reflect non-lysosomal, $Ca^{2+}$-independent, energy-dependent proteolysis (Tawa et al, 1992; Baracos et al, 1995).

### Assay of cathepsins L and B

Animals were killed by cervical dislocation and the gastrocnemius and soleus muscles were removed, trimmed completely of any connective tissue and infringing muscle and washed twice with 10 ml of 250 mM sucrose, 2 mM EGTA, 2 mM EDTA and 20 mM Tris HCl, pH 7.4. The muscles were then individually homogenized in 1 ml of 250 mM sucrose, 2 mM EGTA, 2 mM EDTA, 20 mM Tris HCl, pH 7.4, containing 0.2% Triton X-100, followed by sonication. The supernatants formed after centrifugation at 18 000 *g* for 15 min were used to determine cathepsin activity.

For cathepsin L, the incubation mixture contained 0.1% Brij 35 in water (495 µl), (5 µl) supernatant, 340 mM sodium acetate, 60 mM acetic acid, pH 5.5, 4 mM EDTA and 8 mM dithiothreitol (250 µl), and was preincubated for 5 min at 30°C before adding the substrate *N*-CBZ-PHE-ARG-7-amido-4-methylcoumarin (5 nmoles in 250 µl). After 10 min at 30°C, the reaction was terminated by addition of 1 ml of 100 mM trichloroacetic acid, 100 mM sodium hydroxide, 30 mM sodium acetate and 70 mM acetic acid, pH 4.3. The fluorescence of the free aminomethylcoumarin was determined at an excitation wavelength of 370 nm and an emission wavelength of 430 nm.

Cathepsin B was assayed in a similar manner using a buffer composed of 352 mM potassium dihydrogen phosphate and 48 mM disodium hydrogen phosphate, pH 6.0, 4 mM EDTA, 8 mM cysteine and *N*α-CBZ-Arg-Arg-7-amido-4-methylcoumarin (2.5 nmol in 250 µl) as substrate. The reaction was conducted for 10 min at 40°C and terminated with 1 ml of 100 mM sodium chloroacetate, 30 mM sodium acetate and 70 mM acetic acid, pH 4.3. One unit of enzyme activity is defined as that amount which catalyses the formation of 1 pmol of product from substrate during the 10 min incubation period.

### RNA isolation and Northern blot analysis

Total RNA was extracted from gastrocnemius and soleus muscles, and heart and liver using the acid guanidinium isothiocyanate/phenol/chloroform–isoamylalcohol method (Chomezynski and Sacchi, 1987) and quantitated by absorbance at 260 nm.

The RNA was denatured by heating at 65°C for 10 min and samples (10 µg) were subjected to electrophoresis on 1.2% agarose gels containing 0.67% formaldehyde and transferred onto a Genescreen membrane (NEN Research Products, MA, USA) overnight by capillary action. The RNA was cross-linked to the

© Cancer Research Campaign 1998

**Table 1** Effect of PIF on total body weight and the wet weights of tissues and organs 24 h after administration[a]

| | Control (g) | PIF (g) | P-value[b] |
|---|---|---|---|
| Body weight change | + 0.13 ± 0.11 | −1.60 ± 0.42 | ≤0.01 |
| Spleen | 0.131 ± 0.023 | 0.048 ± 0.018 | ≤0.01 |
| Kidney | 0.196 ± 0.009 | 0.191 ± 0.012 | NS |
| Heart | 0.121 ± 0.025 | 0.156 ± 0.014 | NS |
| Liver | 0.828 ± 0.028 | 0.916 ± 0.029 | ≤0.05 |
| Soleus muscle | 0.006 ± 0.0002 | 0.005 ± 0.0003 | ≤0.005 |
| Gastrocnemius muscle | 0.132 ± 0.023 | 0.048 ± 0.018 | ≤0.01 |

[a]Results are expressed as means ± s.e.m. for five mice per group. [b]Statistical analysis was performed using the unpaired Student's t-test.

**Table 2** The effect of inhibition of lysosomal function with methylamine on protein degradation in soleus muscle of control mice (C) and those bearing the MAC16 tumour (T)

| Addition | Protein degradation (μmol tyrosine g⁻¹ 2 h⁻¹) | | |
|---|---|---|---|
| | C | T | P-value[a] |
| None | 422 ± 34 | 695 ± 3 | 0.001 |
| Methylamine | 433 ± 28 | 554 ± 31 | 0.03 |
| P-value[a] | NS | 0.01 | |

[a]Statistical analysis was performed using the unpaired Student's t-test. NS, not significant.

**Table 3** Activity of lysosomal cathepsins in muscle from control mice (C) and those bearing the MAC16 tumour (T)

| Muscle type | Enzyme activity (U mg⁻¹ protein)[a] | | |
|---|---|---|---|
| | C | T | P-value[b] |
| Cathepsin L | | | |
| Gastrocnemius | 10 750 ± 246 | 11 770 ± 502 | NS |
| Soleus | 10 260 ± 1140 | 59 750 ± 3150 | 0.002 |
| Cathepsin B | | | |
| Gastrocnemius | 51.5 ± 17.3 | 401 ± 39 | 0.02 |

[a]Values are expressed as means ± s.e.m. for six mice per group. [b]Statistical analysis was performed using the unpaired Student's t-test. NS, not significant.

**Table 4** The effect of inhibition of $Ca^{2+}$-dependent proteoltic system on protein degradation in soleus muscle of control mice (C) and those bearing the MAC16 tumour (T)

| Addition | Protein degradation (μmol tyrosine g⁻¹ 2 h⁻¹)[a] | | |
|---|---|---|---|
| | C | T | P-value[a] |
| Calcium chloride | 395 ± 26 | 741 ± 29 | 0.001 |
| No $Ca^{2+}$ + inhibitors | 362 ± 15 | 535 ± 5 | 0.003 |
| P-value[a] | NS | 0.01 | |

[a]Statistical analysis was performed using the unpaired Student's t-test. NS, not significant.

**Table 5** The effect of inhibition of ATP production on protein degradation in soleus muscle of control mice (C) and those bearing the MAC16 tumour (T)

| Addition | Protein degradation (μmol tyrosine g⁻¹ 2 h⁻¹)[a] | | |
|---|---|---|---|
| | C | T | P-value[a] |
| None | 521 ± 21 | 674 ± 62 | 0.01 |
| Inhibitors | 497 ± 59 | 496 ± 32 | NS |
| P-value[a] | NS | 0.01 | |

[a]Statistical analysis was performed using the unpaired Student's t-test. NS, not significant.

membrane using a Spectrolinker XL-1000 UV cross-linker (120 000 mJ cm⁻²) and the membrane allowed to dry. To check the integrity of the RNA and to ensure proper transfer, bands were visualized under UV light after staining with ethidium bromide.

Prehybridization was performed in 50% formamide, 10% dextran sulphate, 1 M sodium chloride, 0.2% bovine serum albumin (BSA), 0.2% polyvinylpyrolidone, 0.2% Ficoll, 0.1% sodium pyrophosphate, 1% sodium dodecyl sulphate (SDS) and 50 mM Tris HCl, pH 7.5, together with 100 μg ml⁻¹ of denatured salmon sperm DNA at 42°C overnight. Radiolabelled probes for E2 (Wing and Banville, 1994) and C9 (Kumatori et al, 1990) together with glyceraldehyde-3-phosphate dehydrogenase (GAPDH) as internal standard were prepared using the Amersham Megaprime DNA labelling system according to the manufacturer's instructions. Hybridization was performed at 42°C, overnight, in the same buffer as for prehybridization in the presence of denatured labelled probes (2.2 × 10⁶ d.p.m. per 30 μl). The membrane was washed twice with 30 mM trisodium citrate and 300 mM sodium chloride (2 × SSC buffer) followed by incubation twice for 1 h with SSC – 1% SDS and a final wash with 0.1 × SSC for 5 min at room temperature. The filters were scanned with a Packard Instant Imager system and finally subjected to autoradiography.

### Statistical analysis

Values are presented as means ± s.e.m. Differences from control values were determined by Student's t-test.

### RESULTS

The changes in total body weight and the weight of some individual organs 24 h after administration of the PIF to female NMRI mice are shown in Table 1. Whereas control animals showed a small weight increase (0.7%), there was a significant ($P \leq 0.01$) decrease in overall body weight (8.6%) in treated animals. The effect on the tissues and organs was variable. Thus, there was a significant decrease in the weight of the soleus and gastrocnemius muscles and spleen, no change in the weight of the heart and kidney, but an increase in weight of the liver (Table 1).

To determine the mechanism for the selective depletion of skeletal muscle in cachexia, a comparison has been made on the loss of soleus muscle induced by PIF and the MAC16 tumour. The effect of blocking lysosomal function with methylamine, an inhibitor of lysosomal acidification, on protein degradation induced by the MAC16 tumour was investigated (Table 2). There was an increased catabolism of soleus muscle proteins in mice

© Cancer Research Campaign 1998

**Table 6** The effect of inhibition (I) of lysosomal, $Ca^{2+}$-dependent and ATP-dependent proteolytic pathways on protein degradation in control (C) and mice treated with PIF (T)[a]

| Type of proteolysis | Protein degradation (μmol tyrosine $g^{-1}$ 2 $h^{-1}$)[b] | | |
|---|---|---|---|
| | C | T | P-value |
| Lysosomal | | | |
| (–I) | 177 ± 16 | 228 ± 10 | 0.05 |
| (+I) | 153 ± 14 | 204 ± 14 | 0.05 |
| P-value | NS | NS | |
| $Ca^{2+}$-dependent | | | |
| (–I) | 234 ± 8 | 268 ± 20 | 0.05 |
| (+I) | 192 ± 13 | 249 ± 16 | 0.05 |
| P-value | NS | NS | |
| ATP-dependent | | | |
| (–I) | 250 ± 13 | 302 ± 20 | 0.05 |
| (+I) | 238 ± 10 | 214 ± 25 | NS |
| P-value | NS | 0.05 | |

[a]Protein degradation was measured in soleus muscle 24 h after i.v. injection of PBS (C) or PIF (T). [b]Values are expressed as means ± s.e.m. for five mice per group. Differences between groups with (+L) or without (–L) the inhibitor were determined by Student's *t*-test.

**Table 7** Effect of a monoclonal antibody (Ab) to PIF on protein degradation in soleus muscle of mice bearing the MAC16 tumour[a]

| Group | Protein degradation (μmol tyrosine $g^{-1}$ 2 $h^{-1}$) |
|---|---|
| MAC16 | 823 ± 82 |
| MAC16 + Ab | 482 ± 29 |
| P-value | 0.01 |

[a]Mice losing weight bearing the MAC16 tumour were randomized to receive either no treatment or a monoclonal antibody to PIF administered i.p. at 0.4 mg b.d. for 48 h. During this time mice receiving no treatment lost 1.3 ± 0.4 g while those receiving antibody lost 0.9 ± 0.7 g.

bearing the MAC16 tumour, and methylamine caused a significant decrease in overall proteolysis in soleus muscle of tumour-bearing mice but not in non-tumour-bearing controls. Thus, there is a rise in the lysosomal proteolytic process in muscles of mice bearing the MAC16 tumour. This was confirmed by measurement of the level of the lysosomal enzymes cathepsins L and B. There was an elevation of cathepsin L in the soleus muscle from mice bearing the MAC16 tumour (Table 3) and an increase in cathepsin B in gastrocnemius muscle. This suggests that a significant proportion of the increased proteolysis was due to an elevation of these lysosomal enzymes.

To determine whether the increase in protein degradation was also due to activation of the non-lysosomal calcium-dependent pathway, soleus muscles from tumour-bearing and non-tumour-bearing mice were incubated under conditions known to block calpains I and II and in the absence of calcium. There was a significant reduction in the rate of protein degradation in the soleus muscle of mice bearing the MAC16 tumour but not in the non-tumour-bearing control group (Table 4), suggesting that the calcium-dependent pathway also contributed to protein degradation in skeletal muscle of mice bearing the MAC16 tumour.

To study the role of the ATP-dependent pathway in protein degradation in soleus muscle induced by the MAC16 tumour, muscles were depleted of ATP after blocking lysosomal and calcium-dependent proteolytic pathways. Under these conditions, protein degradation in non-tumour-bearing animals was not significantly reduced, whereas protein degradation in tumour-bearing mice was significantly reduced (Table 5). There was no significant difference in protein degradation between tumour-bearing and control muscles in the presence of inhibitors. Under these conditions, the ATP content of the muscles was reduced by an average of 73%. Thus, an ATP-dependent non-lysosomal pathway appears to play a major role in the excessive proteolysis of skeletal muscle in mice bearing the MAC16 tumour.

To investigate whether this was dependent on ubiquitin, gastrocnemius muscle from mice bearing the MAC16 tumour was homogenized and the soluble protein was subjected to electrophoresis, immunoblotted and probed with an antibody that specifically recognized ubiquitin-conjugated proteins. As reported by

**Figure 1** (**A**) Ubiquitin–protein conjugates in gastrocnemius muscle of non-tumour-bearing mice (lanes 1–3), mice treated for 24 h with PIF (lanes 4–6) and mice bearing the MAC16 tumour (lanes 7–9). (**B**) Scanning densitometry of the blot in (**A**) expressed in arbitrary units. Results are expressed as means ± s.e.m. and differences from non-tumour-bearing animals are expressed as *, $P<0.05$, as determined using the unpaired Student's *t*-test

**Figure 2** (**A**) Northern blot of gastrocnemius muscle from non-tumour-bearing mice (lanes 1–4). mice bearing the MAC13 tumour (lanes 5–8) and mice bearing the MAC16 tumour (lanes 9–11) 14 days after tumour transplantation. The blots were probed for expression of the 14-kDa ubiquitin carrier protein E2. (**B**) Scanning densitometry of the blot in (**A**) expressed in arbitrary units for non-tumour-bearing mice (open boxes). mice bearing the MAC13 tumour (hatched boxes) and mice bearing the MAC16 tumour (solid boxes). Results are expressed as means ± s.e.m. and differences from non-tumour-bearing animals are expressed as **. $P$<0.01 and ***. $P$<0.001. as determined using the unpaired Student's $t$-test.

**Figure 3** (**A**) Northern blot of gastrocnemius muscle from non-tumour-bearing mice (lanes 1–4). mice bearing the MAC13 tumour (lanes 5–8) and mice bearing the MAC16 tumour (lanes 9–11) 14 days after tumour transplantation. The blots were probed for expression of the C9 proteasome subunit. (**B**) Scanning densitometry of the blot in (**A**) expressed in arbitrary units for non-tumour-bearing mice (open boxes). mice bearing the MAC13 tumour (hatched boxes) and mice bearing the MAC16 tumour (solid boxes)

other workers (Wing et al. 1995). diffuse staining by the antibody was detected in the high molecular mass region (100–200 kDa) of the gel (Figure 1). Scanning densitometry of the blot showed a 42% elevation in the amount of the high molecular mass conjugates of ubiquitin in the soluble gastrocnemius muscle proteins from mice bearing the MAC16 tumour. as well as a significant elevation in PIF-treated mice (Figure 1). This suggests that the ATP–ubiquitin-dependent proteolytic pathway was activated. Because ubiquitin has roles other than in proteolysis (St John et al. 1986). the mRNA encoding other components of the pathway was investigated. Equal amounts of RNA from gastrocnemius muscles of non-tumour-bearing mice. mice bearing the non-cachexia-inducing MAC13 tumour and the MAC16 tumour were compared by Northern hybridization analysis. The RNA blots were probed with the cDNA of the 14-kDa ubiquitin carrier protein E2. which either ligates the ubiquitin directly to the target protein or does so in the presence of ubiquitin protein ligase. Two mRNA transcripts of 1.2 and 1.8 kb were detectable with this probe. arising from different sites of polyadenylation (Figure 2). Although there was only a small change in the level of expression of the 1.8 kb transcript (26% increase). there was a large (twofold) increase in the expression of the 1.2 kb transcript in gastrocnemius muscle of mice bearing the MAC16 tumour and only a small increase in muscle from mice bearing the MAC13 tumour. The RNA blots were also probed for the C9 subunit of the 20S proteasome. the proteolytic core of the 26S proteasome which degrades ubiquitin conjugates (Hershko and Ciechanover. 1992) (Figure 3). This showed a threefold increase in the 1.3 kb transcript in the gastrocnemius muscle of mice bearing the MAC16 tumour. with no significant change in muscle from mice bearing the MAC13 tumour. Control experiments showed that the level of mRNA for GAPDH. a housekeeping gene unrelated to protein breakdown. was similar in all three groups of mice (results not shown). confirming that the differences in mRNA for steps in the ATP–ubiquitin-dependent proteolytic pathway in skeletal muscle of mice bearing the MAC16 tumour were not due to non-specific changes in the levels of all mRNA transcripts.

The effect of PIF on protein degradation in soleus muscle is shown in Table 6. In this case. only the ATP-dependent non-lysosomal pathway was activated. with no contribution from either the

 © Cancer Research Campaign 1998

lysosomal or calcium-dependent pathways. This result suggests that the ATP-dependent pathway is the primary event in the degradation of skeletal muscle by PIF.

To determine whether PIF was responsible for activation of protein degradation in skeletal muscle of mice bearing the MAC16 tumour, mice that were losing weight were treated with a monoclonal antibody for PIF (Todorov et al, 1996*b*) and the extent of protein degradation in soleus muscle was determined. The results presented in Table 7 show an attenuation of the enhanced tyrosine release in muscles from mice bearing the MAC16 tumour treated with antibody. This confirms that PIF is responsible for loss of skeletal muscle in cachectic mice bearing the MAC16 tumour.

### DISCUSSION

Loss of muscle mass during the process of cachexia in mice bearing the MAC16 tumour is associated with the appearance of a PIF in the circulation, which we have shown to be a sulphated glycoprotein of molecular weight 24 kDa (Todorov et al, 1997). Evidence that this material is responsible for the protein degrading activity in serum has been provided with the use of a monoclonal antibody to the 24 kDa glycoprotein, which was capable of neutralization of biological activity in vitro (Todorov et al, 1996*b*). Furthermore, the PIF appears to be responsible for wasting of skeletal muscle in mice bearing the MAC16 tumour, because the polyunsaturated fatty acid, eicosapentaenoic acid (EPA), which we have shown to significantly reduce protein degradation in vivo (Beck et al, 1991), also attenuated protein degradation in vitro initiated by PIF (Lorite et al, 1997). As the PIF induces protein degradation in isolated skeletal muscle, it suggests that the in vivo effects could arise directly without the intervention of other mediators. Indeed, administration of PIF to non-tumour-bearing mice elicits many changes similar to those seen in cachexia, without a depression in food and water intake. Cytokine-mediated effects are characterized by a drop in food and water intake (Matthys and Billiau, 1997), thus distinguishing the action of PIF from the cytokines. In addition to a marked decrease in overall body weight (8.6% in 24 h), there were differential effects on the various tissues and organs reminiscent of the effects of some tumours. Thus, PIF induced a decrease in weight of both gastrocnemius and soleus muscles, although having no effect on the heart and kidney and an increase in weight of the liver. This suggests that the action of PIF is mediated predominantly on skeletal muscle.

Rats transplanted with the Yoshida ascites hepatoma show similar changes in the weight of the gastrocnemius muscle, liver and heart (Baracos et al, 1995), and show activation of proteolytic pathways in skeletal muscle similar to those observed in the MAC16 tumour in the present study. Thus in both models, protein degradation arose from an increase in both a lysosomal and an ATP-dependent proteolytic pathway, but in the Yoshida model there was no contribution from the calcium-dependent proteolytic system. Skeletal muscle contains at least two energy-dependent proteolytic systems, one of which is dependent on ubiquitin (Fagan et al, 1987) and the other independent (Fagan and Waxman, 1989). The former system has been suggested to be mainly responsible for muscle atrophy in Yoshida sarcoma-bearing rats (Temparis et al, 1994) and those bearing the Yoshida ascites hepatoma (Baracos et al, 1995), and appears to be of major importance in mice bearing the MAC16 adenocarcinoma. An increase in the ATP–ubiquitin-dependent proteolytic system in gastrocnemius muscles of mice bearing the MAC16 tumour was supported by the finding of increased levels of ubiquitin-conjugated proteins of high molecular mass. Such large conjugates have been found to be preferentially degraded by the 26S proteasome complex in vitro (Hershko and Ciechanover, 1992). The accumulation of ubiquitin conjugates suggests an increased flux of proteins through the pathway and that the hydrolysis by the 26S proteasome becomes rate limiting (Baracos et al, 1995). Northern blot analysis showed an up-regulation of the mRNA for the 14-kDa E2 involved in substrate ubiquitylation and the rate-limiting step in the ubiquitin conjugation pathway (Wing and Banville, 1994). The 1.2-kb transcript in skeletal muscle has been reported to increase threefold after 2 days of fasting without a significant change in the 1.8-kb transcript (Wing and Banville, 1994). In animals bearing the MAC16 tumour, there was also a twofold increase in expression of the 1.2-kb transcript in gastrocnemius muscle and a small (26%) increase in the 1.8-kb transcript, although there was no reduction in food intake (Beck and Tisdale, 1987). An enhanced production of proteasomes also seems likely from the threefold rise in the mRNA for the proteasome subunit C9.

The ATP-dependent, but not the lysosomal, pathway was also activated in soleus muscle of non-tumour-bearing mice 24 h after administration of PIF, although experiments have not been performed to date to investigate whether this is the ubiquitin-dependent pathway. However, it is likely that PIF contributes directly to the activation of this pathway in skeletal muscle of mice bearing the MAC16 tumour, because antibodies to PIF attenuate the degradation of skeletal muscle proteins. There are other potential mediators such as tumour necrosis factor α (TNF-α) and interleukin 6 (IL-6), which could act in vivo. TNF-α has been shown to produce an increase in ubiquitin gene expression in vitro after incubation for 180 min, although there was no change in the expression of the C8 proteasome subunit, which may require a longer time interval for induction (Llovera et al, 1997). IL-6 has also been found to increase the activity of the 26S proteasome in murine $C_2C_{12}$ myotubes in vitro (Ebisui et al, 1995). However, our previous studies (Mulligan et al, 1992) have failed to find any evidence for an involvement of either TNF-α or IL-6 in the process of cachexia induced by the MAC16 tumour, suggesting that PIF may be directly involved. Further studies are required to dissect out the molecular pathways involved in protein degradation.

### ACKNOWLEDGEMENTS

We thank Mr M Wynter for assistance with the tumour transplantation and Dr W Field for purification of tumour extracts. MJL gratefully acknowledges receipt of a research studentship from the BBSRC.

### REFERENCES

Baracos VE, De Vivo C, Hoyle DHR and Goldberg AL (1995) Activation of the ATP–ubiquitin-proteasome pathway in skeletal muscle of cachectic rats bearing a hepatoma. *Am J Physiol* **268**: E996–E1006

Barrett AJ, Kembhari AA, Brown MA, Kirchke H, Knight GG, Tamai M and Hanada K (1982) L-Trans-epoxysuccinyl-L-leucylamido(4-guanidino)butane (E-64) and its analogues as inhibitors of cysteine proteases including cathepsins B, H and L. *Biochem J* **201**: 189–197

Beck SA and Tisdale MJ (1987) Production of lipolytic and proteolytic factors by a murine tumor-producing cachexia in the host. *Cancer Res* **47**: m5919–5923

Beck SA and Tisdale MJ (1989) Nitrogen excretion in cancer cachexia and its modification by a high fat diet in mice. *Cancer Res* **49**: 3800–3804

Beck SA, Smith KL and Tisdale MJ (1991) Anticachectic and antitumour effect of eicosapentaenoic acid and its effect on protein turnover. *Cancer Res* **51**: 6089–6093

© Cancer Research Campaign 1998

Bird JW. Carter JH. Triemer RE. Brooks RM and Spanier AM (1980) Proteinases in cardiac and skeletal muscle. *Fed Proc* **39**: 20–25
Carmichael MJ. Clague MG. Keir MJ and Johnston ID (1980) Whole body protein turnover. synthesis and breakdown in patients with colorectal carcinoma. *Br J Surg* **67**: 736–739
Chomczynski P and Sacchi N (1987) Single step method of RNA isolation by acid guanidinium thiocyanate–phenol–chloroform extraction. *Anal Biochem* **162**: 156–159
Ebisui C. Tsujinaka T. Morimoto T. Kan K. Iijima S. Yano M. Kominami E. Tanaka K and Monden M (1995) Interleukin-6 induces proteolysis by activating intracellular proteases (cathepsins B and L. proteasome) in $C_2C_{12}$ myotubes. *Clin Sci* **89**: 431–439
Fagan JM and Waxman L (1989) A novel ATP-requiring protease from skeletal muscle that hydrolyzes non-ubiquitinated proteins. *J Biol Chem* **264**: 17868–17872
Fagan JM. Waxman L and Goldberg AL (1987) Skeletal muscle and liver contain a soluble ATP + ubiquitin-dependent proteolytic system. *Biochem J* **243**: 335–343
Hershko A and Ciechanover A (1992) The ubiquitin system for protein degradation. *Annu Rev Biochem* **61**: 761–807
Kumatori A. Tanaka K. Tamura T. Fujiwara T. Ichihara A. Tokunaga F. Onikura A and Iwanaga S (1990) cDNA cloning and sequencing of component C9 of proteasomes from rat hepatoma cells. *FEBS Lett* **264**: 279–282
Llovera M. Garcia-Martinez C. Agell N. Marzabal M. Lopez-Soriano FJ and Argiles JM (1994) Ubiquitin gene expression is increased in skeletal muscle of tumour-bearing rats. *FEBS Lett* **338**: 311–318
Llovera M. Garcia-Martinez C. Agell N. Lopez-Soriano FJ and Argiles JM (1997) TNF can directly induce the expression of ubiquitin-dependent proteolytic system in rat soleus muscles. *Biochem Biophys Res Commun* **230**: 238–241
Lorite MJ. Cariuk P and Tisdale MJ (1997) Induction of muscle protein degradation by a tumour factor. *Br J Cancer* **76**: 1035–1040
Lowell BB. Ruderman NB and Goodman MN (1986) Evidence that lysosomes are not involved in the degradation of myofibrillar proteins in rat skeletal muscle. *Biochem J* **234**: 237–240
Lundholm K. Holm G and Schersten T (1978) Insulin resistance in patients with cancer. *Cancer Res* **38**: 4665–4670
Matthys P and Billiau A (1997) Cytokines and cachexia. *Nutrition* **13**: 763–770
Medina R. Wing SS and Goldberg AL (1995) Increase in levels of polyubiquitin and proteasome mRNA in skeletal muscle during starvation and denervation atrophy. *Biochem J* **307**: 631–637
Melville S. McNurlan MA. Calder AG and Garlick PJ (1990) Increased protein turnover despite normal energy metabolism and responses to feeding in patients with lung cancer. *Cancer Res* **50**: 1125–1131
Mitch WE. Medina R. Grieber S. May RC. England BK. Price SR. Bailey JL and Goldberg AL (1994) Metabolic acidosis stimulates muscle protein degradation by activating the adenosine triphosphate-dependent pathway involving ubiquitin and proteasomes. *J Clin Invest* **93**: 2127–2133
Mulligan HD. Mahony SM. Ross JA and Tisdale MJ (1992) Weight loss in a murine cachexia model is not associated with the cytokines tumour necrosis factor-α or interleukin-6. *Cancer Lett* **65**: 239–243
Nixon DW. Heymsfield SB and Cohen AE (1980) Protein calorie undernutrition in hospitalized cancer patients. *Am J Med* **68**: 683–690
Shamberger RJ (1984) Cancer cachexia. In *Nutrition and Cancer*. p. 353. Plenum Press: London
Smith KL and Tisdale MJ (1993) Mechanism of muscle protein degradation in cancer cachexia. *Br J Cancer* **68**: 314–318
St John T. Gallatin WM. Siegelman M. Smith HT. Fried VA and Weisman IL (1986) Expression cloning of a lymphocyte homing receptor cDNA: ubiquitin is the reactive species. *Science* **231**: 845–850
Tawa NE. Kettlehut IC and Goldberg AL (1992) Dietary protein deficiency reduces lysosomal and non-lysosomal ATP-dependent proteolysis in muscle. *Am J Physiol* **263**: E317–E325
Temparis S. Asensi M. Taillandier D. Aurousseau E. Larbaud D. Obled A. Béchet. D. Ferrara M. Estrela JM and Attaix D (1994) Increased ATP–ubiquitin-dependent proteolysis in skeletal muscles of tumor-bearing rats. *Cancer Res* **54**: 5568–5573
Tiao G. Fagan JM. Samuels N. James JH. Hudson K. Lieberman M. Fischer JE and Hasselgren P-O (1994) Sepsis stimulates non-lysosomal. energy-dependent proteolysis and increases ubiquitin mRNA levels in rat skeletal muscle. *J Clin Invest* **94**: 2255–2264
Todorov P. Cariuk P. McDevitt T. Coles B. Fearon K and Tisdale M (1996*a*) Characterization of a cancer cachectic factor. *Nature* **379**: 739–742
Todorov PT. McDevitt TM. Cariuk P. Coles B. Deacon M and Tisdale MJ (1996*b*). Induction of muscle protein degradation and weight loss by a tumor product. *Cancer Res* **56**: 1256–1261
Todorov PT. Deacon M and Tisdale MJ (1997) Structural analysis of a tumor-produced sulfated glycoprotein capable of initiating muscle protein degradation. *J Biol Chem* **272**: 12279–12288
Waalkes TP and Udenfriend SA (1957) A fluorometric method for the estimation of tyrosine in plasma and tissues. *J Lab Clin Med* **50**: 733–736
Waxman L (1981) Calcium activated proteases in mammalian tissues. *Methods Enzymol* **80**: 664–680
Wing SS and Goldberg AL (1993) Glucocorticoids activate the ATP–ubiquitin-dependent proteolytic system in skeletal muscle during fasting. *Am J Physiol* **264**: E668–E676
Wing SS and Banville D (1994) 14-kDa ubiquitin-conjugating enzyme structure of the rat gene and regulation upon fasting and by insulin. *Am J Physiol* **267**: E39–E48
Wing SS. Haas AL and Goldberg AL (1995) Increase in ubiquitin-protein conjugates concomitant with the increase in proteolysis in rat skeletal muscle during starvation and atrophy denervation. *Biochem J* **307**: 639–645

© Cancer Research Campaign 1998